# Long‐Term Follow‐Up of Patients in a Prospective Study of NA Discontinuation Identifies Different Patterns of HBsAg Loss

**DOI:** 10.1111/apt.70332

**Published:** 2025-08-21

**Authors:** Simon J. Hume, Samuel Hall, Gareth Burns, Sara Vogrin, Daniel Tassone, Paul Desmond, Dilip Ratnam, Miriam T. Levy, Rohit Sawhney, Amanda Nicoll, Zina Valaydon, Simone I. Strasser, Meng Ngu, Marie Sinclair, Chris Meredith, Gayle V. Matthews, Kumar Visvanathan, Jacinta A. Holmes, Alexander J. Thompson

**Affiliations:** ^1^ St Vincent’s Hospital Melbourne Melbourne Victoria Australia; ^2^ University of Melbourne Melbourne Victoria Australia; ^3^ Victorian Infectious Diseases Reference Laboratory Melbourne Victoria Australia; ^4^ Western Health Melbourne Victoria Australia; ^5^ Monash Health Melbourne Victoria Australia; ^6^ Monash University Melbourne Victoria Australia; ^7^ Liverpool Hospital, New South Liverpool New South Wales Australia; ^8^ University of New South Wales Sydney New South Wales Australia; ^9^ Eastern Health Melbourne Victoria Australia; ^10^ Royal Prince Alfred Hospital Sydney New South Wales Australia; ^11^ Concord Hospital Sydney New South Wales Australia; ^12^ Austin Health Melbourne Victoria Australia; ^13^ Bankstown‐Lindcombe Hospital Sydney New South Wales Australia; ^14^ St Vincent’s Hospital Sydney Sydney New South Wales Australia

**Keywords:** alt flare, chronic hepatitis b, functional cure, hbsag level, hbsag loss, hepatitis flare, long‐term follow‐up, nucleos(t)ide analogue discontinuation

## Abstract

**Background and Aims:**

Discontinuing nucleos(t)ide analogues (NAs) may lead to functional cure (HBsAg loss) in selected patients with chronic hepatitis B (CHB). We evaluated the rates and predictors of HBsAg loss during long‐term follow‐up in a prospective cohort.

**Methods:**

This real‐world extension study followed participants from a prospective trial of NA discontinuation. All patients had HBeAg‐negative CHB without cirrhosis. Efficacy outcomes (including HBsAg loss and decline) and safety outcomes [including hepatitis flare and hepatocellular carcinoma (HCC)] were evaluated.

**Results:**

Amongst 97 participants (85% Asian), with a median follow‐up of 7 years, the cumulative incidence of HBsAg loss was 10%, 13% and 22% at 5, 7 and 9 years after stopping NA. HBsAg loss was associated with a lower end‐of‐treatment (EOT) HBsAg level (HR = 0.28, *p* < 0.001), older age (HR = 1.14, *p* = 0.005) and peak off‐treatment HBV DNA level (OR = 0.50, *p* = 0.002). Participants with EOT HBsAg level ≤ 10 IU/mL experienced early HBsAg loss (< 96 weeks) without ALT flares whilst those with EOT HBsAg level ≥ 10 IU/mL experienced late (≥ 96 weeks) HBsAg loss, often following ALT flares (5/8 cases). No cases of hepatic decompensation, liver transplantation or death occurred. Median liver stiffness did not increase. HCC was diagnosed in three individuals (4.4/1000 person‐years).

**Conclusion:**

The rate of functional cure increased during long‐term follow‐up but remained low. EOT HBsAg strongly predicted the likelihood and timing of HBsAg loss. ALT flares were associated with HBsAg decline, and in some cases, with delayed HBsAg loss.

**Trial Registration:** The clinical study was supported by the National Health and Medical Research Council of the study clinical trial ID is NCT02581033

## Graphical abstract

We evaluated long‐term outcomes following nucleos(t)ide analogue discontinuation in HBeAg‐negative chronic hepatitis B. HBsAg loss was infrequent. Early HBsAg loss occurred in patients with low end‐of‐treatment HBsAg levels and without ALT flare, whilst late HBsAg loss typically followed ALT flares and was associated with higher end‐of‐treatment HBsAg levels.

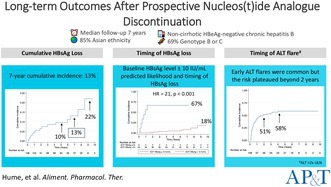

**Abbreviations**
APASL: Asian Pacific Association for the Study of the Liver
cccDNA: Covalently closed circular DNA
CHB: Chronic hepatitis B
HBeAg: Hepatitis B e‐antigen
HBsAg: Hepatitis B surface antigen
HBV: Hepatitis B virus
HCC: Hepatocellular carcinoma
IQR: Interquartile range
LSM: Liver stiffness measurement
MASLD: Metabolic‐associated steatotic liver disease
NA: Nucleos(t)ide analogue

## 1 Introduction

Chronic HBV infection remains a significant global health burden, with over 250 million people affected worldwide [1]. The mainstay of treatment for chronic HBV (CHB) involves the long‐term administration of NAs, which effectively suppress viral replication and reduce the risk of liver disease progression, including cirrhosis and HCC [2, 3, 4]. However, functional cure, defined as sustained loss of hepatitis B surface antigen (HBsAg) with or without seroconversion to anti‐HBs, remains rare despite prolonged therapy.

Over the past decade there has been growing interest in the concept of intentional discontinuation of NA therapy, especially in patients who have achieved long‐term virological suppression and achieved HBeAg seroconversion. This strategy, commonly referred to as a ‘stop strategy’ challenges the traditional paradigm of indefinite NA therapy by exploring whether treatment cessation could trigger immune reconstitution and potentially achieve functional cure in selected patients. The stop strategy offers other potential benefits, including cost reduction, decreased medication burden, and avoidance of long‐term side effects [3]. However, increased monitoring is required; patients are at risk of hepatitis flares and liver decompensation, particularly in the setting of advanced liver disease [5].

Several studies have evaluated the outcomes of stopping NA therapy, particularly in HBeAg‐negative patients [6, 7, 8, 9, 10, 11, 12, 13]. These studies have demonstrated that a subset of patients can achieve durable off‐therapy responses, including functional cure. However, the reported rates of HBsAg loss vary widely [14]. Recent prospective studies suggest that the overall likelihood of HBsAg loss is low in the first 2 years after stopping NA [6, 9]. We have previously performed a prospective, multicentre study that evaluated clinical outcomes to week 96 after stopping NA therapy. The overall rate of HBsAg loss at week 96 of follow‐up was 6% [9]. It is therefore important to be able to identify clinical predictors of HBsAg loss. The most established predictors of HBsAg loss are lower end‐of‐treatment (EOT) HBsAg level and Caucasian compared to Asian ethnicity [3, 6, 7, 9]. There are limited prospective data that describe long‐term outcomes after stopping NA therapy. It is important to understand whether rates of HBsAg loss increase over time, and whether the factors that predict for late loss of HBsAg are the same as those that predict for early HBsAg loss after stopping NA.

A key question is whether hepatitis flares that predict HBsAg loss can be identified, that is, whether hepatitis flares can be therapeutic. This potential benefit must be weighed against the risk of liver fibrosis progression and cirrhosis, liver decompensation and HCC. In our primary study, hepatitis flares were common but were not associated with HBsAg loss at week 96 [9]. However, HBsAg loss after hepatitis flare has previously been reported to be delayed [13, 15, 16]. Moreover, it remains unclear if re‐treatment of a flare with NA influences any possible therapeutic responses [10, 17].

In this manuscript, we report the clinical outcomes after long‐term follow‐up amongst participants enrolled in our prospective study of NA discontinuation amongst individuals living with HBeAg‐negative CHB without cirrhosis. We hypothesised that the incidence of HBsAg loss and decline would increase over time. We sought to identify the characteristics of individuals who achieved late HBsAg loss and examined the influence of ALT flare on HBsAg decline. We also reported on key safety outcomes including fibrosis progression and HCC, which may require longer‐term follow‐up to appreciate.

## 2 Methods

### 2.1 Study Design

This was a real‐world extension study of participants previously enrolled in a prospective multicentre cohort study that evaluated the clinical outcomes at 96 weeks following NA discontinuation amongst non‐cirrhotic participants living with HBeAg‐negative CHB [9]. During the primary study, participants were reviewed 4‐weekly for the first 12 weeks, 6‐weekly from week 12 to 24 and then 12‐weekly until 96 weeks. The protocol criteria for re‐starting NA treatment in the primary study were (i) serum HBV DNA > 2000 IU/mL and serum ALT > 5× ULN for ≥ 16 weeks or ALT > 10× ULN for ≥ 8 weeks; (ii) clinical evidence of hepatic decompensation defined by INR ≥ 1.5 or serum bilirubin level > 2× ULN or ascites or hepatic encephalopathy; or (iii) investigator discretion. Beyond 96 weeks, participants returned to standard of care follow‐up including HCC surveillance according to guidelines [4]. Clinical decision‐making, including decisions regarding re‐treatment, was made at the discretion of the treating physician.

Pathology testing, including liver function tests, HBV serology, HBsAg level and HBV DNA, was performed at the local laboratory of each site. Serum HBV DNA was measured using the COBAS Ampliprep/COBAS TaqMan platform (Roche Diagnostics, lower limit of detection 20 IU/mL) at all sites. Serum HBsAg level was measured using the Elecsys HBsAg II quant II (Roche Diagnostics) with a lower limit of reporting of 0.05 IU/mL. Clinical and laboratory data were collected from the electronic medical record. The reference range for ALT was set as per the local laboratory. Participants underwent a Fibroscan for liver stiffness measurement (LSM) at EOT and at the end of follow‐up. Fibroscan assessments were conducted by trained operators using standardised protocols. All measurements adhered to manufacturer‐recommended quality criteria, including at least 10 valid measurements and an IQR to median ratio ≤ 30%.

### 2.2 Participants

Key inclusion criteria for the primary study were (i) continuous NA therapy for at least 2 years with HBV DNA levels < 20 IU/mL for at least 18 months; (ii) HBeAg‐negative chronic hepatitis B at the time NA therapy was initiated; (iii) HBsAg‐positive, HBeAg‐negative and anti‐HBe‐positive at the time of screening; and (iv) absence of cirrhosis, defined as liver stiffness < 9.6 kPa at screening and, if available, liver histology showing METAVIR stage ≤ F3 prior to NA initiation. Exclusion criteria included (i) HBeAg positivity at the time NA therapy was started; (ii) a diagnosis of cirrhosis at any time, including histology showing METAVIR stage F4 prior to NA initiation, a previous clinical diagnosis of cirrhosis or a history of liver decompensation; (iii) median liver stiffness > 9.5 kPa at screening; (iv) a history of hepatocellular carcinoma; (v) HBV‐associated extrahepatic manifestations; (vi) co‐infection with HIV, hepatitis C virus or hepatitis D virus; (vii) concomitant immunosuppression; and (viii) significant alcohol consumption (> 30 g/day for women and > 50 g/day for men).

**FIGURE 1:**
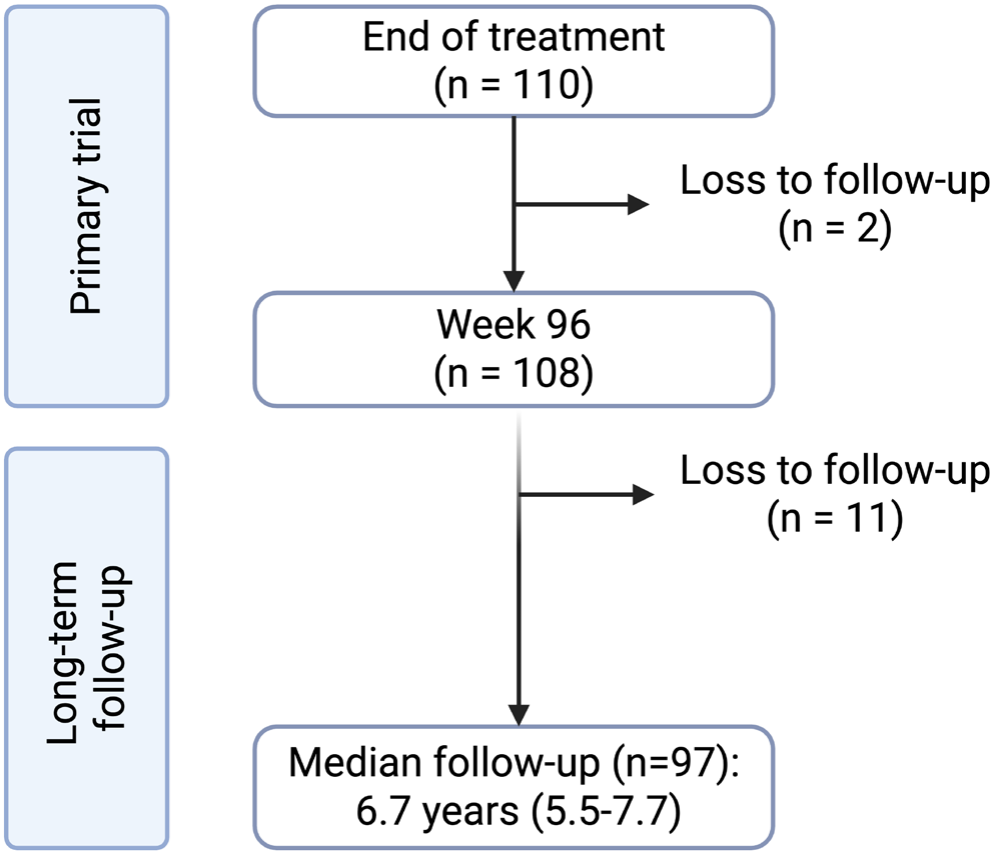
Study flow diagram.

Of the 110 participants enrolled in the primary study, two were lost to follow up very early (week 4 and 12, respectively) and therefore were excluded from all analysis (Figure 1). No long‐term follow‐up data was available for 11 participants, and therefore these participants were only included for the purposes of survival analysis (censored at time of loss to follow up).

### 2.3 Clinical Endpoints

The primary endpoints of interest were the incidence of HBsAg loss and HBsAg decline [considered both as a categorical variable (±1 log_10_ reduction in HBsAg) and as a continuous variable]. HBsAg loss was defined by undetectable quantitative HBsAg (< 0.05 IU/mL) as well as negative qualitative HBsAg serology. We also examined the rates of ALT flare [ALT > 2× upper limit of normal (ULN)]. ALT flares were stratified by severity: mild flare (ALT 2‐5× ULN), moderate flare (ALT 5‐10× ULN) and major flare (> 10× ULN). Virological reactivation was defined by the occurrence of detectable HBV DNA following NA discontinuation, and virological relapse was defined by HBV DNA ≥ 2000 IU/mL. Immune control was defined as being off‐treatment and having a HBV DNA < 2000 IU/mL and ALT < 2× ULN. Other key safety data was also reported, including hepatic decompensation, change in LSM (as measured by Fibroscan), HCC diagnosis and death or liver transplant. In addition to LSM, changes in serum‐based estimators of fibrosis were also reported. AST to platelet ratio index (APRI) was calculated as (AST ÷ ULN of AST) × 100 ÷ platelet count (10^9^/L); Fibrosis‐4 (FIB‐4) was calculated as (age [years] × AST [U/L]) ÷ (platelet count [10^9^/L] × √ALT [U/L]). The modified PAGE‐B (mPAGE‐B) score was calculated to predict the risk of HCC, incorporating patient age, sex, platelet count and serum albumin with weighted points assigned to each variable as previously described, yielding a maximum score of 21 [18].

### 2.4 Statistical Analysis

Descriptive data were presented as median [interquartile range (IQR) or frequency (percentage)]. Logarithmic (log_10_) units were used to assess the change in HBsAg level over time and to quantify HBV DNA. Log_10_ values for results < 1 were set to 0 to avoid negative values. Associations between baseline (EOT) characteristics and clinical outcomes over time were examined with Kaplan–Meier analysis and Cox proportional hazards regression, with results expressed as a hazard ratio (HR) with 95% CI. Patients were censored at the time of last follow‐up. For the analysis of ALT flares, only the first flare per subject was considered, and participants were additionally censored at the time of HBsAg loss or re‐treatment (since participants were no longer at risk of flare after either of these events). Associations between off‐treatment characteristics and clinical outcomes were examined with logistic regression, with results expressed as odds ratio (OR) with 95% CI. When evaluating the incidence of a ≥ 1 log HBsAg decline from EOT, participants who achieved a transient 1 log_10_ decline but in whom the HBsAg decline at the end of follow‐up was < 1 log_10_ were counted as not having achieved the outcome.

The two‐tailed Fisher's exact test and Mann–Whitney tests were used for comparison of categorical and continuous variables between groups at end of follow‐up. Variables with a *p*‐value < 0.2 on univariable analysis were subsequently included in a multivariable model to account for potential confounders. The Wilcoxon signed‐rank test was used to compare paired measurements. Changes in HBsAg level and LSM over time were reported as the median (IQR) of the paired difference of the cohort. *P*‐values of < 0.05 by two‐tailed test were considered statistically significant. Statistical analysis was performed using Stata 17.0 software.

### 2.5 Ethics Statement

This study was approved by the St Vincent's Hospital Ethics Committee (ID Number: 032/14) and by the institutional review boards at all participating sites. It was conducted in accordance with the ethical principles outlined in the 1975 Declaration of Helsinki and applicable local regulations. Informed consent was obtained from all participants prior to inclusion, and confidentiality was maintained throughout the study.

## 3 Results

### 3.1 Participant Characteristics

The primary study included data from 108 participants with follow‐up to week 96; 97 of these participants were followed with data collection beyond week 96 (Figure 1). Long‐term outcomes were not available beyond week 96 in 11 participants, for whom analysis of clinical outcomes was censored at week 96. The median follow‐up time (*n* = 97) was 6.7 years (IQR 5.5–7.7; range 4.3–9.1) with a total of 668 person‐years. There were no differences in the baseline characteristics between the 11 participants who were lost to follow up at 96 weeks and those who remained in long‐term follow‐up, Table S1.

As previously reported, most participants were of Asian ethnicity and the most common genotype was genotype B (Table 1). The median HBsAg level (IU/mL) at EOT was 680 (195–1819), and all participants were non‐cirrhotic. Nine (9%) participants had an EOT HBsAg level ≤ 10 IU/mL. Six participants had achieved HBsAg loss (6%) before week 96 of follow‐up [9].

**TABLE 1:** Baseline (end‐of‐treatment) characteristics of participants with long‐term follow‐up.

Baseline characteristics	*n* = 97^a^
**Male, *n* (%)**	55 (57%)
**Age, median (IQR) years**	56 (50–63)
**Treatment, *n* (%)** Entecavir Tenofovir Adefovir/lamivudine Lamivudine	61 (63%) 29 (30%) 4 (4%) 3 (3%)
ALT, median (IQR) U/L	26 (18–33)
**Ethnicity, *n* (%)** Asian Caucasian Other	82 (85%) 11 (11%) 4 (4%)
**Genotype, *n* (%)** A B C D E Not available	4 (4%) 46 (47%) 21 (22%) 14 (14%) 2 (2%) 10 (10%)
HBsAg level, median (IQR) IU/mL	680 (195–1819)
≤ 10	9 (9%)
10–100	7 (7%)
100–1000	39 (40%)
> 1000	42 (43%)
Liver stiffness measurement, median (IQR) kpa	4.6 (3.6–5.5)

^a^ There were no differences in the baseline characteristics between the 11 participants who were lost to follow up at 96 weeks and those who remained in long‐term follow‐up; Table S1.

### 3.2 HBsAg Loss

**FIGURE 2:**
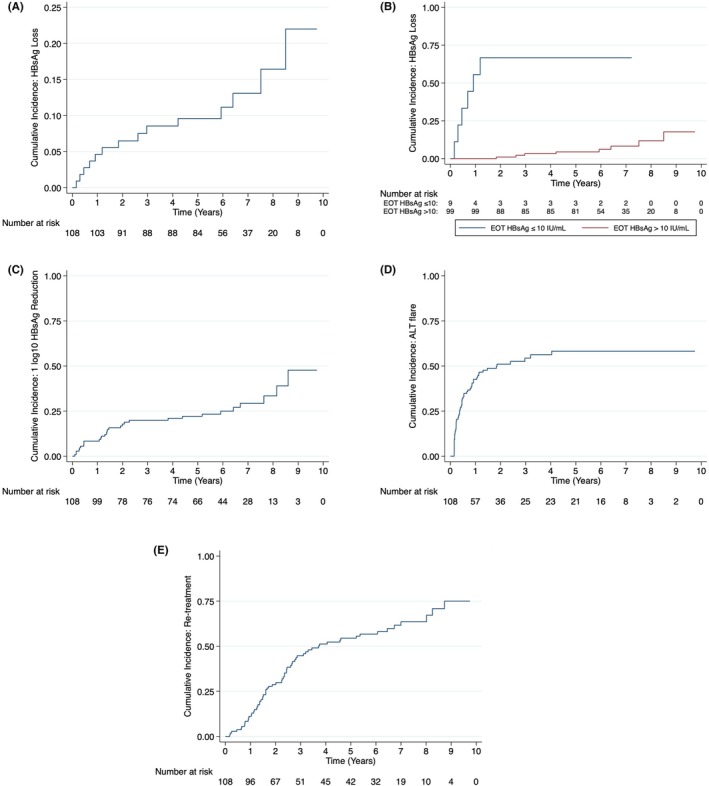
Cumulative incidence of (A) HBsAg loss, (B) HBsAg loss stratified by EOT HBsAg level, (C) ≥ 1 log_10_ HBsAg reduction, (D) ALT flare, (E) Re‐treatment.

HBsAg loss was observed in eight participants ≥ 96 weeks (individual patient plots: Figure S1A–H). The cumulative incidence of HBsAg loss was 9.6%, 13.1% and 22.0% at 5‐, 7‐ and 9‐year follow‐ups following NA discontinuation (Figure 2A). The median time from EOT to HBsAg loss was 2.1 years (IQR 1.8–5.9 years; range 57 days—8.5 years). HBsAg loss was durable and there were no instances of HBsAg seroreversion. 12/14 (86%) achieved anti‐HBs seroconversion with a median titre of 77 mIU/mL (19–202) at the end of follow‐up.

The strongest predictor of HBsAg loss was low HBsAg level at EOT. We previously identified EOT HBsAg level ≤ 10 IU/mL as a key threshold for identifying HBsAg loss <96 weeks of follow‐up [9]. Those with EOT HBsAg level ≤ 10 IU/mL had a higher chance of HBsAg loss compared to the remainder of the cohort (*n* = 6/9 (67%); HR = 21.1, CI 6.7–66.5, *p* < 0.001). EOT HBsAg ≤ 100 IU/mL also predicted HBsAg loss, but 6/7 (86%) of patients in this group who achieved HBsAg loss had an EOT HBsAg level ≤ 10 IU/mL (Figure S2). The 7‐year cumulative incidence of HBsAg loss for those with an EOT HBsAg level ≤ 10 IU/mL and ≤ 100 IU/mL was 67.67% and 37.00%. Amongst participants with EOT HBsAg level ≤ 10 IU/mL who achieved HBsAg loss (*n* = 6/6), HBsAg loss occurred <96 weeks after stopping NA (median 24 weeks, range 8–62 weeks) and in the absence of an ALT flare or NA re‐treatment. Further HBsAg loss did not occur amongst this group during long‐term follow‐up (Figure 2B). Amongst the 3 participants who had EOT HBsAg ≤ 10 IU/mL and who remained HBsAg‐positive, one individual maintained HBeAg‐negative infection (peak HBV DNA ≤ 3.1 log_10_ IU/mL) during long‐term follow‐up, and two individuals re‐started NA therapy in the context of peak serum HBV DNA levels of 4.9 and 6.5 log_10_ IU/mL, respectively. The end of follow‐up HBsAg levels (IU/mL) for the three patients was 0.45, 0.26 and 5.90, respectively. No participant with EOT HBsAg level > 10 IU/mL achieved HBsAg loss before week 96.

HBsAg loss ≥ week 96 was observed in eight participants (Figure 2B), all of whom had an EOT HBsAg level > 10 IU/mL [median (IQR) 517 (222–1139), range 63–2143]. Amongst participants with EOT HBsAg level > 10 IU/mL, the cumulative incidence of HBsAg loss was 3%, 4%, 8% and 18% at 3, 5, 7 and 9 years after stopping NA therapy. Participants with EOT HBsAg level > 10 IU/mL who achieved late HBsAg loss commonly experienced virological relapse [median peak HBV DNA 4.5 (3.6–6.1) log_10_ IU/mL] and associated ALT flares [5/8, 63%; median peak ALT 173 (127–1857) IU/mL] prior to HBsAg loss, Figure S3. 4/8 (50%) were re‐treated with NA before HBsAg loss. In addition, one of the two participants who achieved HBsAg loss without a predefined ALT flare (> 2 x ULN) experienced a 3.7‐fold increase in off‐treatment ALT compared to EOT. Amongst those who experienced HBsAg loss following an ALT flare, the median time from peak ALT to HBsAg loss was 3.4 years (3.1–6.5). Amongst those who were re‐treated, the time from re‐starting NA treatment to HBsAg loss was 0.5, 2.5, 3.9 and 5.8 years, respectively.

The only other baseline characteristic that was associated with HBsAg loss was older age (Table 2). Those aged over 60 years at EOT had a higher chance of HBsAg loss (HR = 3.8 (1.4–11.1); *p* = 0.014, Figure S4). There was no association between other baseline patient characteristics and HBsAg loss over time (Table 2).

**TABLE 2:** Association between patient characteristics and likelihood of (i) HBsAg loss and (ii) ≥ 1 log reduction in HBsAg.

	HBsAg loss		≥ 1 log reduction in HBsAg
Baseline characteristics (end‐of‐treatment)			
	HR (95% CI), univariable^a^	HR (95% CI), multivariable	HR (95% CI), Univariable^e^
**Male**	1.80 (0.60–5.73), *p* = 0.324		1.56 (0.73–3.33), *p* = 0.255
**Age**	1.14 (1.03–1.20), *p* = 0.005*	1.10 (1.01–1.19), *p* = 0.023*	1.03 (0.99–1.07), *p* = 0.092
**Treatment** Entecavir Tenofovir Other	Ref 1.30 (0.40–4.34), *p* = 0.705 3.76 (0.95–14.85), *p* = 0.059	1.03 (0.27–4.01), *p* = 0.970 2.60 (0.64–10.63), *p* = 0.185	Ref 1.06 (0.48–2.36), *p* = 0.887 1.44 (0.42–5.00), *p* = 0.563
**ALT**	0.98 (0.93–1.04), *p* = 0.576		1.02 (0.98–1.05), *p* = 0.292
**Ethnicity** Asian Caucasian Other	Ref 2.22 (0.61–8.10), *p* = 0.226 2.46 (0.31–19.40), *p* = 0.391		Ref 1.53 (0.59–4.01), *p* = 0.384 n/a^c^
**Genotype** B or C Other	Ref 1.75 (0.52–5.83), *p* = 0.363		Ref 1.59 (0.61–4.17), *p* = 0.343
**HBsAg level** (log_10_)	0.28 (0.16–0.47), *p* < 0.001*	0.28 (0.16–0.50), *p* < 0.001*	0.80 (0.57–1.12), *p* = 0.188
**Liver stiffness measurement**	1.53 (1.11–2.10), *p* = 0.009*	1.51 (0.99–2.28), *p* = 0.051	1.13 (0.87–1.47), *p* = 0.339

Off‐treatment clinical characteristics			
	OR (95% CI), Univariable^b^	OR (95% CI), multivariable^#^	OR (95% CI), univariable
**Peak off‐treatment HBV DNA**			Ref
< 4 log_10_ IU/mL ≥ 4 log_10_ IU/mL	Ref 0.08 (0.02–0.30), *p* < 0.001*	Ref 0.14 (0.03–0.61), *p* = 0.009*	Ref 0.99 (0.34–2.91), *p* = 0.991
**Flare** (ALT, x ULN)			
< 2x (No flare)	Ref	Ref	Ref
≥ 2x (ALT flare)	0.38 (0.12–1.25), *p* = 0.113	1.53 (0.33–7.10), *p* = 0.587	2.22(0.89–5.57), *p* = 0.089
<2x (No flare)	Ref		Ref
2‐5x (Mild) 5‐10x (Moderate) (Major)	0.44 (0.11–1.78), *p* = 0.246 n/a^d^ 0.33 (0.07–166), *p* = 0.178		1.57 (0.44–5.64) 0.76 (0.14–4.08) 4.10 (1.40–11.99), *p* = 0.010*
**NA re‐treatment**	0.10 (0.03–0.41), *p* = 0.001*	0.20 (0.05–0.89), *p* = 0.034*	0.68 (0.28–1.66), *p* = 0.395

* *p* < 0.05.

^a^ Univariable Cox proportional hazards regression (95% CI) of the overall cohort, censored at time of loss to follow up (additional censoring for re‐treatment and HBsAg loss for the outcome of ALT flare).

^b^ Univariable logistic regression of participants with long‐term follow‐up.

^c^ There were no instances of 1 log HBsAg reduction in the ‘other’ (non‐Asian and non‐Caucasian) ethnicity group; therefore, this group was combined with the Caucasian group.

^d^ There were no instances of HBsAg loss amongst the moderate flare group (ALT 5‐10x); therefore, this group was combined with the mild flare group.

^e^ Multivariable analysis was not performed because only one baseline and one off‐treatment variable met the pre‐specified threshold of *p* < 0.2 in univariable analysis.

After stopping treatment, a significant rise in HBV DNA levels was a negative predictor for HBsAg loss. A higher off‐treatment peak in HBV DNA (log_10_ IU/ml) was associated with a lower chance of HBsAg loss (OR = 0.5, CI 0.3–0.6; *p* = 0.002). Participants whose off‐treatment HBV DNA rose to > 4 log_10_ IU/mL after stopping NA had a very low chance of HBsAg loss (5/77 (6%) vs. 9/20 (45%); OR = 0.08, CI 0.02–0.30; *p* < 0.001). This association persisted after adjustment for EOT HBsAg level, age and flare status (presence/absence of ALT flare >5x ULN) [adjusted OR = 0.20, CI 0.02–0.47, *p* = 0.004]. Of the 77 participants who experienced hepatitis flare accompanied by a marked off‐treatment rise in HBV DNA (≥ 4 log10 IU/mL), 58 (75%) were re‐treated. Of the 19 participants who remained off‐ treatment, end of follow‐ up outcomes were as follows: HBsAg loss (*n* = 3, 16%), HBV DNA < 2000 IU/mL (*n* = 10, 53%) and HBV DNA ≥ 2000 IU/mL (*n* = 6, 32%).

Overall, participants who achieved HBsAg loss were less likely to have been re‐treated [3/14 (21%) vs. 11/14 (79%), *p* < 0.001]. Amongst participants who experienced ALT flare (ALT >2x ULN), HBsAg was more common in non‐re‐treated patients compared to re‐treated patients [3/9 (33.33%) vs. 2/45 (4.44%), *p* = 0.028]. The proportion of patients who were re‐treated and achieved HBsAg loss <96 weeks, HBsAg loss >96 weeks, and no HBsAg loss was 0/6 (0%), 4/8 (50%) and 60/83 (72%). No other off‐treatment characteristic, including flare status, was associated with HBsAg loss (Table 2).

### 3.3 HBsAg Level Reduction

There was a significant reduction in HBsAg levels observed over time in this cohort. The median HBsAg levels (IU/mL) at EOT, week 96, and end of follow‐up were 680 (195–1819), 309 (60–1010) and 122 (3–594) (*p* < 0.001). The median matched pair absolute and relative reduction in HBsAg levels from EOT to end of follow‐up were 349 IU/mL (113–1153) and 74% (47–95), respectively. The 5‐ and 7‐year cumulative incidences of HBsAg < 100 IU/mL were 39% and 54%; and of HBsAg < 10 IU/mL were 20% and 27% (Figure S5). The cumulative incidence of ≥ 1 log10 HBsAg decline was 22% at 5 years and 29% at 7 years (Figure 2C).

**FIGURE 3:**
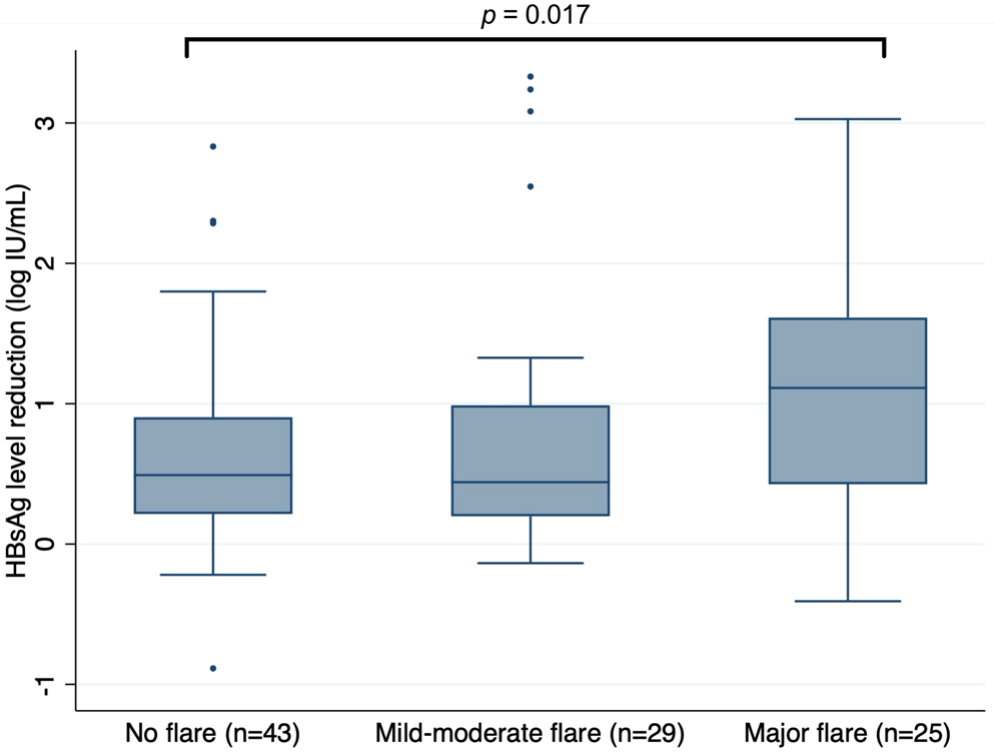
Box plots comparing the median HBsAg reduction from end of treatment to end of follow‐up, stratified by flare status. *P*‐value is derived from the paired comparison (Mann–Whitney test) of change in HBsAg (log10 IU/mL) between those who experienced a major flare vs. the remainder of the cohort.

Participants who experienced a major flare were more likely to achieve a ≥ 1 log_10_ HBsAg reduction compared to those who did not experience any ALT flare (OR = 4.10, CI 1.40–11.99; *p* = 0.010). Peak off‐treatment ALT (log_10_ U/L) was also associated with ≥ 1log_10_ HBsAg decline (OR = 2.7, CI 1.2–6.0; *p* = 0.013). Paired comparisons of HBsAg decline over time demonstrated similar results: there was a greater median decline in HBsAg level amongst participants who experienced a major flare [1.1 (0.4–1.6)] compared to no ALT flare at all [0.5 (0.2–0.8)]; *p* = 0.017 (Figures 3 and S6). No other characteristic, including age, genotype, ethnicity, peak off‐treatment HBV DNA or NA re‐treatment (yes/no), was associated with a > 1 log_10_ reduction in HBsAg (Table 2).

### 3.4 Virological Relapse and ALT Flare

Most episodes of virological relapse and/or ALT flare occurred during the first 96 weeks of follow‐up. The rates of virological relapse and ALT flare up to week 96 have previously been reported [9]. In brief, virological reactivation and virological relapse were experienced by 96/97 (99%) and 87/97 (90%) of participants up to week 96. ALT flare (> 2x ULN) was common and occurred early [median time to flare was 5 (2–11) months]. The 96‐week cumulative incidence of any ALT flare was 52% and major flare was 22%, respectively [9]. The 3‐ and 5‐year cumulative incidence of ALT flare was 54% and 58% (Figure 2D). The cumulative incidence of major flare (ALT > 10 x ULN) at 3 and 5 years was 23% and 26% (Figure S7). The median time (days) from the onset of the first ALT flare (ALT 2x ULN) to re‐treatment for all ALT flares was 298 (103–510). Amongst participants who experienced an ALT flare, 47 (87%) had a single flare and 7 (13%) had two discrete flares during follow‐up. No participant experienced more than two discrete flares.

We considered separately the 44 participants who remained HBsAg‐positive and who were not on NA therapy at week 96 and entered long‐term follow‐up. 4/44 (9%) experienced an ALT flare during the third year of follow‐up (Figure 2D). Another 4 participants (9%) experienced an ALT flare during long‐term follow‐up beyond year 3 (one was a major flare and occurred in year 5). A higher peak off‐treatment HBV DNA (which occurred within 90 days prior to flare for 6/8 (75%)) was the only characteristic associated with the occurrence of an ALT flare beyond 96 weeks (data not shown).

Prior tenofovir treatment and non‐Asian ethnicity were associated with the risk of ALT flare on multivariable analysis (Table S2). Participants who stopped tenofovir (vs. entecavir) experienced ALT flare earlier [median 2.2 (1.8–3.7) vs. 7.4 (5.0–13.7) months; *p* < 0.001] and experienced more overall ALT flares (3‐year cumulative incidence: 72% vs. 47%; 5‐year cumulative incidence: 78% vs. 50%; HR = 2.68, CI 1.6–4.6; *p* < 0.001) (Figure S8A). Asian ethnicity was associated with a reduced risk of flare (HR = 0.48 CI 0.3–0.9; *p* = 0.021). Whilst participants with EOT HBsAg level ≤ 10 (vs. >10 IU/mL) experienced less ALT flares by end of follow‐up [1/9 (11%) vs. 53/88 (60%), *p* = 0.010] (Figure S8B and Table S2), this association was not statistically significant on time‐to‐event analysis. The 7‐year cumulative incidence of ALT flare for those with an EOT HBsAg level ≤ 10 IU/mL and ≤ 100 IU/mL was 12.50% and 60.22%.

### 3.5 Disease Phase and Re‐Treatment Status at End of Follow‐Up

At the end of follow‐up, 14/97 (14%) had achieved HBsAg loss, 15/97 (15%) were in long‐term immune control, 8/97 (8%) had a HBV DNA > 2000 IU/mL but normal serum ALT off‐treatment and the remaining 60/97 (62%) had achieved virological suppression after re‐commencing NA therapy (Figure 2E and Table S3). All participants re‐treated by week 96 (*n* = 33) met pre‐specified criteria for a severe and/or prolonged hepatitis flare [9]. The reasons for re‐treatment beyond 96 weeks (*n* = 31) were ALT>ULN + HBV DNA > 2000 IU/mL (58%), ALT<ULN + HBV DNA > 2000 IU/mL (32%), patient preference (6%) and new diagnosis of HCC (3%). Of the four patients who achieved HBsAg loss after re‐treatment, three participants discontinued NA therapy successfully without seroreversion and one continued due to a diagnosis of HCC. All other participants who were re‐treated remained on NA therapy at the end of follow‐up. Overall, the proportion of patients who were re‐treated amongst those who experienced any ALT flare and major ALT flare was 45/54 (83%) and 24/25 (96%), respectively. All participants who were re‐treated achieved virological suppression.

Amongst those in immune control at end of follow‐up, 12/15 (80%) had already achieved immune control by 96 weeks. Achieving immune control by end of follow‐up was associated with younger age and a lower EOT LSM at EOT (Table S4). None of the participants who remained off‐treatment at the end of follow‐up met treatment‐naïve criteria for treatment initiation (all had HBV DNA < 2000 IU/mL or ALT<ULN) [4].

### 3.6 Safety

Safety to week 96 has previously been reported [9]. In brief, bilirubin rose > 2× ULN in 5 participants before week 96 in the context of a hepatitis flare (major hepatitis flare in 4/5; moderate hepatitis flare in 1/5). One of these patients was admitted to hospital for further investigation; however, the others were managed as outpatients. Three of these patients were started on NA therapy. The serum bilirubin levels fell to normal rapidly in all 5 participants with resolution of the hepatitis flare. There were no liver decompensation events (INR > 1.5, hepatic encephalopathy, ascites) up to week 96.

Beyond 96 weeks no participant experienced a bilirubin rise to above 2x ULN, and there were no instances of hepatic decompensation, liver transplantation or death. Three participants in total (all HBsAg‐positive at diagnosis) were diagnosed with HCC over the follow‐up period (4.4 cases per 1000 person‐years; Table S5). Two of the three participants had previously been re‐treated for an ALT flare and had undetectable HBV DNA at the time of HCC diagnosis. The third participant had an HBV DNA level of 83 IU/mL at diagnosis. One participant, with comorbid metabolic‐associated steatotic liver disease (MASLD), was diagnosed with cholangiocarcinoma 12 months prior to the diagnosis of HCC. The participants' ages at diagnosis of HCC were 69, 73, and 75 years, respectively. The LSMs measured by Fibroscan at EOT were 2.8, 7.1, and 6.7 kPa, respectively. All three participants had mPAGE‐B scores at diagnosis consistent with high HCC risk (scores of 14, 17, and 15, respectively; high risk defined as ≥ 13 [18]). All were diagnosed with either very early or early‐stage disease (Barcelona Clinic Liver Cancer Stage 0 or A); two were treated with locoregional therapy, and one with surgical resection. All three participants remained in complete HCC remission at the end of follow‐up (7.1, 2.1 and 2.2 years following diagnosis of HCC, respectively). The participant who developed HCC and was treated with surgical resection also achieved HBsAg loss; the pre‐operative HBsAg level was 0.056 IU/mL, and the HBsAg level was undetectable when next evaluated (4 months post‐operatively).

Paired LSMs were available for 92 participants at baseline and end of follow‐up, with a median follow‐up time of 6.6 (5.7–7.9) years. There was no significant change in median LSM between EOT and end of follow‐up [4.6 (3.6–5.5) vs. 4.3 (3.7–5.3); *p* = 0.424]. The median paired change in LSM was 0.2 (−0.8–1.0). There was also no significant change in the median LSM for participants who experienced any ALT flare [0(−0.8–0.8); *n* = 52] and those who experienced a major flare [0 (−0.5–0.6); *n* = 23] (Figure S9). Amongst the small minority (*n* = 19) who experienced a ≥ 1 kPa increase in LSM, 10 (53%) experienced a flare and 9 (47%) did not; *p* = 0.795. There was no change in APRI or FIB‐4 score from EOT to end of follow‐up, including amongst the subgroup who experienced a major flare (Table S6).

## 4 Discussion

We report the long‐term outcomes (median 7 years) after stopping NA therapy of a prospectively enrolled cohort of predominately Asian individuals living with HBeAg‐negative CHB. The rate of HBsAg loss increased over time and was durable. However, the overall rate was low, with an overall cumulative incidence of 13% at 7 years. This rate was similar to the 6‐year cumulative incidence reported in a large real‐world single centre study of Asian patients [8] but lower than what has been described in Caucasian cohorts [12, 19]. Amongst participants with an EOT HBsAg level > 10 IU/mL, the 7‐year cumulative incidence of HBsAg loss was very low at 8%.

We observed two patterns of HBsAg loss which varied according to EOT HBsAg levels. In the small subset of patients with EOT HBsAg level ≤ 10 IU/mL, HBsAg loss was common (cumulative incidence 67%), occurred early (< 96 weeks) and with no ALT flare. In contrast, amongst participants with EOT HBsAg levels > 10 IU/mL, HBsAg loss was rare; but when it occurred, it was typically associated with a preceding ALT flare and was delayed—HBsAg loss occurred > 96 weeks in all, at a median of 3.4 years following peak ALT flare. This difference may explain why studies of varied duration of follow‐up have not consistently shown an association between ALT flare and HBsAg loss [12]. We hypothesise that there may be different biological mechanisms underpinning early vs. late HBsAg loss. The data suggest that a more robust immune response, causing hepatocyte cytolysis, is required to clear the larger liver HBV reservoir present in people with EOT HBsAg level > 10 IU/mL [20, 21]. Even in the absence of HBsAg loss, a major flare (ALT > 10× ULN) was associated with a greater likelihood of achieving a 1 log_10_ reduction in HBsAg levels compared to participants in whom there was no flare. However, the majority of patients who experienced a major flare did not achieve HBsAg loss. Identification of the key immunological pathways involved in this process will be important and may inform novel therapeutic development. For example, we have previously identified an association between peripheral monocyte and natural killer cell TLR signalling and ALT flare in this cohort [22] suggesting a key role for innate immune signalling.

Older age was associated with an increased chance of HBsAg loss (aHR 1.14, *p* = 0.023) and a trend towards a slightly higher chance of achieving a ≥ 1 log10 HBsAg reduction (HR = 1.03, *p* = 0.092). Whilst this finding is in keeping with some existing data [13, 23], it is conflicting with other studies which found that age was either not associated with HBsAg loss [8] or associated with a reduced chance of HBsAg loss [11]. The positive association between age and HBsAg loss may reflect a greater time‐dependent decline in intrahepatic cccDNA during prolonged NA therapy in older patients prior to treatment discontinuation [24].

Previous data, largely retrospective with the risk of selection bias, have suggested that re‐starting NA therapy may be associated with a lower likelihood of achieving HBsAg loss after stopping NA [10, 17, 25]. Indeed, in this prospective cohort, no patients who achieved early HBsAg loss were re‐started on NA therapy. However, as noted, no patient with early HBsAg loss had an indication for re‐starting NA therapy. The key question is whether re‐starting NA therapy in a patient having a major flare reduces the likelihood of HBsAg loss. In this cohort, the majority of participants who achieved late HBsAg loss after an ALT flare did so despite being re‐started on NA therapy. We were not able to specifically test for an association between re‐starting NA treatment and achieving HBsAg loss—24/25 participants experiencing a major flare were re‐started on NA treatment—but it was reassuring that NA re‐treatment did not abort HBsAg loss. Furthermore, the association between major ALT flare and HBsAg decline was independent of NA re‐treatment, again suggesting that NA use does not abort the therapeutic benefit of an ALT flare.

Individuals who stop tenofovir (vs. entecavir) tend to experience flares earlier; but due to the relatively short duration of follow‐up of existing studies, it has remained controversial as to whether the overall rate of flare differs [6, 9]. This was addressed in the current study with long‐term follow‐up demonstrating that whilst the difference reduced over time, participants who stopped tenofovir (versus entecavir) not only experienced flares earlier but also experienced more flares overall.

Long‐term immune control was achieved by 15% of HBsAg‐positive patients at the end of treatment, 80% of whom had experienced immune control by 96 weeks. Re‐treatment rates were high, with 66% of the cohort being recommenced on NA therapy by the end of follow‐up. All individuals who were re‐treated achieved virological suppression.

This study provides reassurance regarding the real‐world long‐term safety of NA discontinuation. ALT flares were common early, but the risk plateaued beyond 96 weeks, suggesting that intensive safety monitoring is most important early after stopping NA. There were no cases of hepatic decompensation, liver transplant or death at any time. Despite the common occurrence of early ALT flares, there was no increase in liver stiffness measurements over a median follow‐up time of 7 years. There were three cases of hepatocellular carcinoma (3% or 4.4 cases per 1000 person‐years) which is in line with what would be expected of a similar on‐treatment cohort of non‐cirrhotic HBeAg‐negative patients with CHB [26]. Moreover, all three cases had an elevated risk as evidenced by high mPAGE‐B scores, and one case had significant co‐factors.

A key strength of our study was the prospective recruitment, detailed characterisation and long‐term retention in a follow‐up period which allowed for the appreciation of increased HBsAg loss and decline over time as well as reassurance regarding the low rates of HCC and lack of fibrosis progression. The overall rate of loss to follow up was low. There were some limitations. The cohort was predominantly Asian and the size of the cohort was relatively small although it is one of the largest cohorts to have been recruited and followed prospectively. In particular, we were not powered to analyse a comparison of HBsAg thresholds of 10 vs. 100 IU/mL for predicting early HBsAg loss, and we were not able to test the relevance of these thresholds to non‐Asian populations. Nevertheless, whilst an HBsAg threshold of < 100 IU/mL has been endorsed by recent guidelines, future study should aim to validate a threshold of < 10 IU/mL as a superior predictor of HBsAg loss amongst Asian patients [27]. Moreover, due to the low proportion of participants who achieved HBsAg loss after experiencing a flare, the study was underpowered to analyse predictors of therapeutic versus non‐therapeutic flares. Finally, there was no control arm, limiting the analysis of HBsAg decline over time relative to the natural history of CHB.

Finally, we propose some recommendations for clinical practice based on our data. We propose that in non‐cirrhotic Asian patients, the small subset of individuals with EOT HBsAg levels < 10 IU/mL are most suitable for the NA‐stop strategy. These patients have a high likelihood of achieving early HBsAg loss, and ALT flare is uncommon. We would not recommend stopping NA therapy for Asian patients who have an HBsAg level > 10 IU/mL outside of clinical trials. In these patients, ALT flare is common, particularly in the first 96 weeks after stopping NA, whilst HBsAg loss is rare and delayed. Although the biology of late HBsAg loss after an ALT flare is interesting, the rate of HBsAg loss is low, and the burden of increased monitoring is significant for patients. Given that re‐treatment does not preclude subsequent HBsAg loss, we suggest early re‐treatment for individuals who experience a hepatitis flare (ALT > 5× ULN), particularly when accompanied by a HBV DNA level ≥ 4 log_10_ IU/mL. We recommend continuing NA therapy in Asian people with HBsAg levels > 10 IU/mL, whilst awaiting novel cure strategies that are in development.

## 5 Conclusions

In conclusion, HBsAg loss increased with long‐term follow‐up but remained low in this predominantly Asian cohort. HBsAg level at EOT was a useful biomarker for identifying the likelihood of HBsAg loss. We identified two different patterns of HBsAg loss, where EOT HBsAg level > 10 was associated with delayed HBsAg loss after ALT flare in a small number of patients. ALT flare was also associated with a decline in HBsAg levels, suggesting that some ALT flares may be therapeutic. The stop strategy was safe but requires close monitoring, particularly amongst Asian patients with HBsAg level > 10 IU/mL.

## Author Contributions

**Simon J. Hume:** conceptualization, writing – original draft, methodology, visualization, writing – review and editing, formal analysis, investigation, data curation. **Samuel Hall:** data curation, writing – review and editing. **Gareth Burns:** data curation, writing – review and editing. **Sara Vogrin:** formal analysis, writing – review and editing. **Daniel Tassone:** data curation, writing – review and editing. **Paul Desmond:** writing – review and editing. **Dilip Ratnam:** writing – review and editing, data curation. **Miriam T. Levy:** data curation, writing – review and editing. **Rohit Sawhney:** data curation, writing – review and editing. **Amanda Nicoll:** writing – review and editing, data curation. **Zina Valaydon:** data curation, writing – review and editing. **Simone I. Strasser:** conceptualization, writing – original draft, methodology, visualization, writing – review and editing, formal analysis, investigation, data curation, data curation, writing – review and editing, data curation, writing – review and editing, formal analysis, writing – review and editing, data curation, writing – review and editing, writing – review and editing, writing – review and editing, data curation, data curation, writing – review and editing, data curation, writing – review and editing, writing – review and editing, data curation, data curation, writing – review and editing, writing – review and editing, data curation, writing – review and editing, data curation, writing – review and editing, data curation, writing – review and editing, data curation, data curation, writing – review and editing, writing – review and editing, investigation, conceptualization, conceptualization, data curation, formal analysis, investigation, methodology, visualization, writing – review and editing, supervision, conceptualization, data curation, formal analysis, investigation, methodology, visualization, writing – review and editing, funding acquisition, supervision. **Meng Ngu:** writing – review and editing, data curation. **Marie Sinclair:** writing – review and editing, data curation. **Chris Meredith:** writing – review and editing, data curation. **Gayle V. Matthews:** data curation, writing – review and editing. **Kumar Visvanathan:** writing – review and editing, investigation, conceptualization. **Jacinta A. Holmes:** conceptualization, data curation, formal analysis, investigation, methodology, visualization, writing – review and editing, supervision. **Alexander J. Thompson:** conceptualization, data curation, formal analysis, investigation, methodology, visualization, writing – review and editing, funding acquisition, supervision.

## Ethics Statement

This study was approved by the St Vincent's Hospital Ethics Committee (ID Number: 032/14) and by the institutional review boards at all participating sites. It was conducted in accordance with the ethical principles outlined in the 1975 Declaration of Helsinki and local regulatory requirements.

## Consent

Informed consent was obtained from all participants prior to inclusion, and confidentiality was maintained throughout the study.

## Conflicts of Interest

The authors declare no conflicts of interest.

## Supporting information


**Data S1:** apt70332‐sup‐0001‐DataS1.docx.

## Data Availability

The data that support the findings of this study are available on request from the corresponding author. The data are not publicly available due to privacy or ethical restrictions.
