# Supplementary material for: Long‐Term Follow‐Up of Patients in a Prospective Study of NA Discontinuation Identifies Different Patterns of HBsAg Loss
Source: Aliment Pharmacol Ther. 2025 Aug 21;63(2):231–41. doi: 10.1111/apt.70332 (PMC12746397; doi:10.1111/apt.70332)

Supplementary Materials

# Tables

## Table 1. Comparison of baseline characteristics and outcomes for the original cohort, compared to those with long-term follow up

| **Characteristic** | **Original Cohort**  **(n = 108)** | **Extension cohort**  **(n= 97)** | **LTFU beyond 96 weeks (n=11)** | **P-value^#^** |
| --- | --- | --- | --- | --- |
| **Baseline Characteristics** | | | | |
| **Male, n (%)** | 62 (57%) | 55 (56%) | 7 (64%) | 0.756 |
| **Age,** median [IQR] years | 56 (50-62) | 56 (50-63) | 53 (51-59) | 0.402 |
| **Treatment**  Entecavir  Tenofovir  Adefovir/lamivudine  Lamivudine | 68 (63%)  32 (30%)  4 (4%)  4 (4%) | 61 (63%)  29 (30%)  4 (4%)  3 (3%) | 7 (64%)  3 (27%)  0 (0%)  1 (9%) | 0.658 |
| **ALT**, median [IQR] U/mL | 25 (18-33) | 26 (18-33) | 24 (22-28) | 0.867 |
| **Ethnicity**  Asian  Caucasian  Other | 91 (84%)  13 (12%)  4 (4%) | 82 (85%)  11 (11%)  4 (4%) | 9 (82%)  2 (18%)  0 (0%) | 0.758 |
| **Genotype**  A  B  C  D  E  Missing | 5 (5%)  47 (44%)  22 (23%)  15 (14%)  2 (2%)  16 (15%) | 4 (4%)  46 (47%)  21 (22%)  14 (14%)  2 (2%)  10 (10%) | 1 (9%)  1 (9%)  2 (18%)  1 (9%)  0 (0%)  6 (55%) | 0.245 |
| **HBsAg level,** median [IQR] IU/mL  ≤ 10  10-100  100-1000  >1000 | 615 (190-1777)  9 (8%)  11 (10%)  44 (41%)  44 (41%) | 680 (195-1819)  9 (9%)  7 (7%)  39 (40%)  42 (43%) | 293 (93-825)  0 (0%)  4 (36%)  5 (45%)  2 (18%) | 0.233 |
| **Liver stiffness measurement by Fibroscan®**, median (IQR) kpa | 4.6 (3.7-5.6) | 4.6 (3.6-5.5) | 4.7 (4.1-6.0) | 0.289 |
| **Outcomes** | | | | |
| **ALT flare (x ULN)**  >2x (ALT flare)  <2x (No flare)  2-5x (Mild flare)  5-10x (Moderate flare)  >10x (Major flare) | 59 (55%)  49 (45%)  20 (19%)  12 (11%)  27 (25%) | 55 (56%)  42 (43%)  18 (19%)  12 (12%)  25 (26%) | 7 (64%)  2 (18%)  0 (0%)  2 (18%) | 0.632 |
| **HBsAg loss, n (%)** | 14 (13%) | 14 (14%) | 0 (0%) | 0.353 |
| **1 log HBsAg decline, n (%)** | 30 (28%) | 29 (30%) | 1 (9%) | 0.284 |

^#^Two-tailed Fisher’s exact or Mann–Whitney test, comparison of those loss to follow up (LTFU) beyond 96 weeks (n=11), compared to those with long-term follow-up (n=97)

## Table 2. Univariable and multivariable Cox proportional hazards regression: Predictors of ALT flare

|  | **HR (95% CI), univariable** | **HR (95% CI), multivariable** |
| --- | --- | --- |
| **Male** sex | 0.86 (0.51-1.43), p=0.565 |  |
| **Age years at EOT** | 1.02 (0.99-1.05), p=0.172 | 1.01 (0.99-1.04), p = 0.314 |
| **Treatment**  Entecavir  Tenofovir  Other | Ref  2.63 (1.53-4.53), p<0.001*  1.14 (0.35-3.75), p = 0.823 | Ref  3.12 (1.67-5.80), p<0.001* |
| **ALT at EOT** | 0.99 (0.97-1.02), p=0.872 |  |
| **Ethnicity**  Asian  Non-Asian | Ref  2.09 (1.12-3.92), p=0.021* | Ref  2.93 (1.02-8.46), p=0.047* |
| **Genotype** B or C  Other | 0.59 (0.32-1.06), p=0.078 Ref | 1.06 (0.41-2.70), p=0.911  Ref |
| **HBsAg level,** log_10_ IU/mL at EOT | 1.12 (0.84-1.52), p=0.421 |  |
| **HBsAg level (IU/mL)**  ≤10  >10 | 0.24 (0.03-1.70), p=0.152  Ref | 0.26 (0.04-1.97), p=0.193 |
| **Liver stiffness measurement, kpa** | 1.03 (0.85-1.26), p=0.745 |  |

## Table 3. Rates of clinical outcomes by end of follow up

| **Outcomes** | **N = 97** |
| --- | --- |
| **Duration of follow up,** median (IQR) years | 6.7 years (5.7-7.8) |
| **HBsAg loss,** n (%) | 14 (14%) |
| **1 log HBsAg decline**, n (%) | 29 (30%) |
| **HBsAg < 100,** n (%)^^^  **HBsAg < 10,** n (%)^^^ | 47 (58%) 24 (27%) |
| **Virological reactivation,** n (%) | 96 (99%) |
| **Peak HBV DNA,** log_10_ IU/mL | 5.3 (4.2-6.5) |
| **ALT flare (x ULN)**  <2x (No flare)  >2x (ALT flare)  2-5x (Mild flare)  5-10x (Moderate flare)  >10x (Major flare) | 43 (44%)  54 (56%)  17 (18%)  12 (12%)  25 (26%) |
| **Immune control** | 15 (15%) |

^As a proportion of patients with EOT HBsAg level ≥100 and ≥10 IU/mL respectively

## Table 4. Association between patient characteristics and achieving immune control at end of follow up

|  | **OR (95% CI),**  **univariable^@^** | **OR (95% CI),**  **multivariable^#^** |
| --- | --- | --- |
| **Male** | 2.38 (0.70-8.08), p=0.166 | 4.18 (0.94-18.65), p=0.060 |
| **Age** | 0.90 (0.84-0.97), p=0.005* | 0.90 (0.82-0.97), p=0.008* |
| **Treatment^#^**  Entecavir  Other^#^ | Ref  0.57 (0.17-1.94), p=0.367 |  |
| **ALT** | 1.00 (0.95-1.06), p=0.841 |  |
| **Ethnicity**  Asian  Non-Asian | Ref  0.35 (0.04-3.86), p=0.325 |  |
| **Genotype**  B or C  Other | Ref 0.30 (0.06-1.41), p=0.126 | Ref  0.21 (0.02-1.97), p=0.172 |
| **HBsAg level** (log_10_) | 1.31 (0.70-2.46), p=0.405 |  |
| **Liver stiffness measurement** | .51 (0.28-0.91), p=0.022* | 0.51 (0.27-0.96), p=0.039* |
|  | **OR (95% CI),**  **Univariable&** | **OR (95% CI),**  **multivariable^#^** |
| **Peak off-treatment HBV DNA**  <4 log_10_ IU/mL  ≥4 log_10_ IU/mL | Ref  0.22 (0.67-0.70), p=0.010* | Ref  0.28 (0.08-1.00), p=0.051 |
| **Flare** (ALT, x ULN)  <2x (No flare)  ≥2x (ALT flare) | Ref  0.34 (0.11-1.07), p=0.066* | Ref  0.51 (0.14-1.82), p=0.305 |
| <2x (No flare)  2-10x (Mild-moderate)  >10x (Major) | Ref  0.53 (.0.15-1.88), p=0.325  0.14 (0.02-1.15), p=0.067 |  |

^*^p<.05
^#^No participant with “other” treatment experienced immune control so the “other” category was combined with the tenofovir category

## Table 5. Characteristics of participants who developed hepatocellular carcinoma

| **Age (at EOT)** | **Sex** | **Genotype** | **EOT HBsAg (IU/mL)** | **Peak ALT (U/L)** | **LSM^#^ (kPa, at EOT)** | **LSM^#^ (kPa, at EOFU)** | **Diagnosis, months from EOT** | **Co-factor** | **BCLA Stage** | **Treatment** | **Current disease status** |
| --- | --- | --- | --- | --- | --- | --- | --- | --- | --- | --- | --- |
| 67 | M | D | 2077 | 2012 | 3.1 | 2.8 | 26 | - | 0 | Radiofrequency ablation | CR^^^ |
| 70 | M | B | 4.31 | 369 | 5.5 | 7.1 | 35 | MASLD^!^ | 0 | Microwave ablation | CR |
| 68 | M | D | 1066 | 1857 | 8.5 | 6.7 | 83 | - | A | Resection | CR |

^#^Liver stiffness measurement by transient elastography

^!^Metabolic associated steatotic liver disease  ^^^Complete response (CR)

## Supplementary Table 6. Changes in APRI and FIB-4 score from EOT to end of follow-up

|  | **Overall cohort** | **No flare** | **Major flare** |
| --- | --- | --- | --- |
| **APRI** | | | |
| End-of-treatment (EOT) | 0.26 (0.20-0.37) | 0.28 (0.19-0.37) | 0.25 (0.22-0.35) |
| End of long-term follow-up | 0.26 (0.21-0.35) | 0.27 (0.19-0.36) | 0.25 (0.20-0.37) |
| **FIB-4** | | | |
| End-of-treatment | 1.28 (1.02-1.56) | 1.37 (1.11-1.54) | 1.25 (1.03-1.54) |
| End of long-term follow-up | 1.28 (1.03-1.53) | 1.36 (1.13-1.53) | 1.25 (1.02-1.55) |

# Figures

## Figure 1. Patient plots of individuals who achieved HBsAg loss ≥ 96 weeks

(A)

(B)

(C)

(D)

(E)

(F)

(G)

(H)

## Supplementary Figure 2. Cumulative incidence of HBsAg loss stratified by EOT HBsAg level: (A) ≤10 vs 10-100 vs > 100 IU/mL (B) ≤100 vs >100 IU/mL

## Supplementary Figure 3. Off-treatment ALT level over time stratified by end of treatment HBsAg level (± 10 IU/mL)

^#^ALT > 10x displayed as 10x ULN

## Supplementary Figure 4. Cumulative incidence of HBsAg loss, stratified by age

## Supplementary Figure 5. Cumulative incidence of HBsAg decline to (A) <100 IU/mL and (B) <10 IU/mL

(A)

(B)

## Figure 6. Box plots comparing the median HBsAg level from end of treatment (EOT) to end of follow up (EOFU), stratified by flare status

Black arrow depicting the median change in HBsAg level from EOT to EOFU for each flare category

P-value is derived from the paired comparison (Mann–Whitney test) of change in HBsAg (log10 IU/mL) between those who experienced a major flare vs the remainder of the cohort

## Supplementary Figure 7. Cumulative incidence of major flare

Censored at re-treatment or HBsAg loss (whichever occurred earlier)

## Figure 8. Cumulative incidence of ALT flare (>2x ULN) stratified by (A) NA therapy type and (B) EOT HBsAg level

(A)

(B)

## Figure 9. Change in liver stiffness measurement by Fibroscan®, stratified by flare status


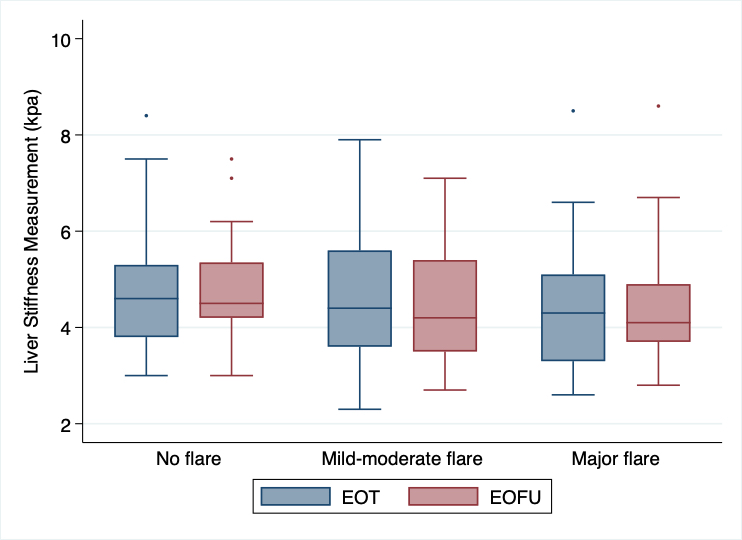

Supplement: Supplementary file 1 — Data S1: apt70332‐sup‐0001‐DataS1.docx. [file APT-63-231-s001.docx]
